# Reproducibility in Cognitive Hearing Research: Theoretical Considerations and Their Practical Application in Multi-Lab Studies

**DOI:** 10.3389/fpsyg.2020.01590

**Published:** 2020-07-16

**Authors:** Antje Heinrich, Sarah Knight

**Affiliations:** ^1^Manchester Centre for Audiology and Deafness, University of Manchester, Manchester, United Kingdom; ^2^Department of Psychology, University of York, York, United Kingdom

**Keywords:** reproducibility, replication, retest reliability, multi-laboratory collaboration, cognitive hearing science, Stroop

## Abstract

In this article, we consider the issue of reproducibility within the field of cognitive hearing science. First, we examine how retest reliability can provide useful information for the generality of results and intervention effectiveness. Second, we provide an overview of retest reliability coefficients within three areas of cognitive hearing science (cognition, speech perception, and self-reported measures of communication) and show how the reporting of these coefficients differs between fields. We argue that practices surrounding the provision of retest coefficients are currently most rigorous in clinical assessment and that basic science research would benefit from adopting similar standards. Finally, based on a distinction between direct replications (which aim to keep materials as close to the original study as possible) and conceptual replications (which test the same purported mechanism using different materials), we discuss new initiatives which address the need for both. Using the example of the auditory Stroop task, we provide practical illustrations of how these theoretical issues can be addressed within the context of a multi-lab replication study. By illustrating how theoretical concepts can be put into practice in empirical research, we hope to encourage others to set up and participate in a wide variety of reproducibility-related studies.

## Introduction

Reproducibility is a core requirement for the accrual of scientific knowledge and the advancement of a field. The concept derives its importance from the fact that in an ideal world we would expect repeated measurement of the same variable to lead to the same result. However, this is often not the case, and it is unclear whether divergent results occur because measurement conditions were not comparable in some essential aspect or because of random error. Differentiating between these two potential explanations has important implications for test selection, experimental setup, and interpretation of results from single studies, as well as for the assessment of scientific progress as a whole.

We acknowledge that a lively discussion exists within the wider field of behavioral science regarding the theoretical question of what exactly constitutes “reproducibility” and how best to assess it (Schmidt, 2009; Goodman et al., 2016). We would like to extend this discussion to the field of cognitive hearing science and suggest ways of incorporating relevant theoretical concepts into empirical practice. Cognitive hearing science is an interdisciplinary field that aims to understand how auditory and cognitive processes combine to allow speech understanding in complex listening environments (Arlinger et al., 2009). Before examining cognitive hearing science specifically, however, we first propose that the following general distinctions are crucial when discussing replication: the *level* of replication and the *type* of replication. In terms of level, two levels of replication exist: individual-level replications, which concern the reproducibility of individual differences, often in the form of retest reliability; and group-level replications, which concern the reproducibility of effect sizes, often expressed as differences between group means. The former refers to the similarity of an individual’s test scores at different points in time when no intervention has been applied. The latter refers to the likelihood that an experimental effect based on group-level differences will replicate. In terms of type, two types of replication exist: direct and conceptual. This distinction follows theoretical analyses by Hendrick (1991) and Schmidt (2009) who, among other things, identified the following two important classes of variables that shape the nature of an experiment: the *primary information focus* and the *contextual background*. The primary information focus of an experiment refers to both the hypothesis to be tested (the *immaterial* focus) and also the instructions, materials, and events experienced by participants (the *material realization*). The contextual background into which the primary information focus is embedded includes participant characteristics, the physical setting, the particular experimenter, minute material differences (called *specific task variables*; e.g., different screen resolutions), and also the procedures for the selection and allocation of participants [which Schmidt (2009) defines as a separate class]. Based on this framework, we define *direct replication* studies as those which aim to keep the material realization of the primary information focus as close to the original study as possible. These studies vary contextual background in an effort to discount sampling error or artifacts as explanations for published findings. *Conceptual replications*, on the other hand, aim to verify the underlying hypotheses of previous studies by constructing an experiment which tests the same purported mechanisms but uses different materials. Thus, conceptual replications address the same immaterial primary information focus, but vary its material realization. Often, type and level of replication vary together. For instance, retest reliability measures tend to involve individual-level scores from direct replications. Conceptual replications, on the other hand, typically involve the replicability of group means, but may also investigate individual-level scores.

It is important to note that these distinctions are not entirely unproblematic. In the case of replication type (direct versus conceptual), the distinction is a matter of degree and centers around questions such as: Up to what point is a replication still direct? When does it become conceptual? Which changes in material, setup or test population are consequential? In the case of replication level (individual versus group), the distinction is conceptually and mathematically clear – but these different types of replication have directly opposing requirements for sample selection and experimental set-up. Experimental studies are based on average responses, which require homogeneous samples in order to reduce unwanted between-subject variability and increase the chance that even small experimental effects will reach a significance threshold. By contrast, correlational studies, which examine phenomena based on individual differences within a population, require large between-subject variability to be most sensitive. Indeed, low between-subject variability in this type of study adversely affects the reliability of individual differences, decreasing the likelihood of replicability for results based on correlations with other factors (Hedge et al., 2018). In other words, a fundamental methodological tension exists between two commonly-used research designs. Being aware of this tension is particularly important when both approaches are combined within a single study, a practice increasingly common in cognitive hearing science (Lunner and Sundewall-Thorén, 2007; Heinrich et al., 2010; Schneider et al., 2016).

In this article, we discuss reproducibility in the context of three types of measures commonly used within cognitive hearing science: behavioral measures of cognition, behavioral measures of speech perception, and self-report measures of communication ability and speech perception. In the following section, we first discuss individual-level replications before turning to group-level replications.

## Replication Level: Individual Differences – the Case of Retest Reliability

Replication of individual differences concerns the stability of a score over time – that is, how likely it is that an individual’s score will replicate when the individual has not undergone any intervention. Typically, such replications are carried out as direct replications (i.e., the same test is performed repeatedly) and the resulting measure of consistency is termed “retest reliability.” Retest reliability provides information about the robustness and precision of a test on the level of individual scores. Such information is crucial in experimental studies because the predictive value of variables is limited by their precision, as measured by reliability (Spearman, 1904; Nunnally, 1970). In clinical diagnosis and prognosis, retest reliability is a prerequisite for accurate assessment and the monitoring of interventions over time. Despite the importance of retest reliability, it is rarely measured and reported, a situation that is not unique to cognitive hearing science (Watson, 2004).

One important question in retest reliability is how to adequately account for measurement error. Error can be systematic – arising in a relatively predictable fashion from sources such as practice effects, fatigue, or use of specific raters – or it may be random. Furthermore, strategies to control for systematic and random error can be employed on a design level and/or at the analysis stage. For example, one common design-level strategy to avoid systematic error arising from memorization of stimulus materials is to use similar but non-identical stimulus lists when tests are re-administered. Strategies at the analysis stage, meanwhile, usually center around the choice of statistical measure. Retest reliability of interval-scaled and normally distributed data is typically assessed using either of the following two parameters: Pearson’s Product-Moment Correlation (PPMC) or the Intraclass Correlation Coefficient (ICC). The PPMC has historically been a popular parameter of retest reliability despite its problems with accounting for systematic error and bias, and its overestimation of reliability (Heise, 1969). The ICC, on the other hand, explicitly estimates both systematic and random error, thereby allowing for a distinction between estimates of agreement and consistency (Aldridge et al., 2017). In this context, agreement refers to the extent to which observed raw scores obtained by a measurement tool for one individual match between raters or time-points in the absence of any actual (systematic) change in the outcome being measured. In contrast, consistency refers to how similar the relative rank of an individual’s score within the group is across raters/times; the actual value of the raw scores itself is unimportant. In addition to estimating retest reliability, the ICC allows the estimation of minimal difference scores. Minimal difference scores are vital in the context of intervention effectiveness, since they indicate the smallest difference that can be considered a significant change (Jacobson and Truax, 1991; Chelune et al., 1993) rather than arising from measurement error. Regardless of the strategy used to control for error, calculated values for retest reliability are typically judged according to the guidance given by Cicchetti and Sparrow (1981): retest reliability below 0.40 is poor, between 0.40 and 0.59 is fair, between 0.60 and 0.74 is good, and 0.75 and above is excellent.

Supplementary Table S1 gives example values of retest reliability scores for a number of tests used in cognitive hearing science. In particular, we focus on the three types of measure mentioned above: behavioral measures of cognition^1^, behavioral measures of speech perception, and self-report measures of communication ability and speech perception. The table gives the name of the test, the number of participants and make-up of the sample on which the retest reliability value is based, the time period between administrations, which type of retest coefficient was used, its value, and whether any systematic differences in means were reported. Providing this information enables the reader to assess for themselves whether systematic error has occurred and how well it was taken into account for a particular estimate.

Note that behavioral tests used for clinical assessment are typically more rigorously assessed for retest reliability than tests used in lab-based research, and speech testing is no exception in this regard. Some clinical speech tests have undergone extensive validation in order to construct equivalent but non-identical forms or stimulus lists, such as the CUNY NTS (Dubno and Dirks, 1982), NU-6 word test (Causey et al., 1983), and the QuickSIN (Killion et al., 2004). Speech tests developed exclusively for research purposes, on the other hand, normally undergo less stringent validation, although there are notable exceptions such as the SPIN-R (Bilger et al., 1984). In fact, there are countless examples, including from our own research, where speech material was newly developed and used to investigate differences between groups of interest without first rigorously testing the accuracy of the material as an outcome measure (Heinrich et al., 2008, 2010; Heinrich and Schneider, 2011; Knight and Heinrich, 2017, 2019). We suggest that lab-based science research would benefit from aspiring to similar standards to clinical assessment when it comes to the measurement and reporting of retest reliability and test validity.

### A Group-Level View of Individual Differences

In addition to examining retest reliability estimates from single studies, it is possible to examine how well retest reliability estimates themselves replicate across different studies – in other words, to take a group-level view of individual-level retest reliability. To give a sense of the insights gained from such an approach, we re-print in Supplementary Table S2 a subset of information from Supplementary Table S1 and provide more detailed descriptions of the studies involved, including sample composition, test administration, and statistical details. Note that retest reliability estimates often vary by about 0.2. For some tests (Letter Number Sequencing test, semantic fluency), it appears unclear which, if any, of the methodological differences caused this disparity. For the Digit Span test, it is possible that the difference in retest reliability values was caused by the varying retest intervals (days versus a year) but it may also be due to other unreported differences between the studies. For phonological fluency, it is troubling to note that, although the participant groups had similar characteristics and the same coefficient was used to estimate retest reliability (PPMC), the estimates still varied between 0.63 and 0.82. An additional concern for the phonological fluency estimates is that the one study which examined differences between the first and second tests found systematic differences. These were then not adequately taken into account, thus possibly leading to an overestimate of retest reliability. As at least one other study also used PPMC estimates without testing for systematic differences, it is possible that other values are overestimated as well (Heise, 1969). Finally, the Trail Making A&B tests were the most frequently replicated. The results from these studies suggest that values around 0.6 may be more representative of this test’s retest reliability in many situations than 0.8 or 0.9.

Methodological variations in testing are likely to explain some of the differences found for retest reliability estimates. However, how much they explain and how much is due to error remains to be established by systematic investigation. There is a clear need for better validation of experimental measures, resulting in more reliable and comparable tests. Such a shift in practices would represent one means of tackling the replication crisis currently facing psychological science in general (Open Science Collaboration, 2015) and most likely cognitive hearing science too. Given that it is not always clear how robust scores are in various populations when they are repeatedly assessed, either using identical tests or comparable but non-identical stimulus lists, it can be difficult to know what results mean and whether interventions and manipulations have had the intended effect. We therefore advocate for retest scores of identical and comparable stimulus lists to be routinely included as standard measurements. This would enable researchers to assess robustness of scores and list equivalence more easily, make more informed choices regarding outcomes measures, and also encourage methods for improving robustness, particularly of non-identical lists.

## Replication Level: Group Effects

Besides individual differences, replication of group differences is another essential aspect of scientific practice. Sometimes, one or two key conditions from a previous study are included in a new study in order to verify the premise of the basic effect (Studdert-Kennedy and Shankweiler, 1970; Cutting, 1974; Amitay et al., 2002; Ziegler et al., 2005)—although these replications do not always yield the intended result (Baker et al., 2008; Arsenault and Buchsbaum, 2016). Publishing complete direct replications (and their failure) has been a longstanding problem, since publication guidelines of scientific journals have traditionally stated that the scope of their publications is innovative, new, or original research. This almost exclusive focus on novelty as practiced by many scientific journals in the past has given rise to a number of concerns. In particular, due to publication and other biases (Ioannidis, 2005; Ioannidis et al., 2014), only positive results tended to be published (Scheel et al., 2020). Such a practice historically made it difficult to explore whether replication failure was due to inadvertent consequential changes in the paradigm or to random error. Additionally, the replication of previous work typically represents a minor focus of a given publication, making it difficult to track the state of replications in a field (see also Rosenthal, 1979).

However, this practice is in the process of changing, with more journals now stating that they value the internal (i.e., within study) and external (i.e., across study) replication of results (e.g., *Journal of Psychology: General; Psychological Science; Royal Society*). The recent change in approach to replication in psychological research can be illustrated by the publication of two large-scale replication projects: the Reproducibility Project undertaken by the Open Science Collaboration (Open Science Collaboration, 2012, 2015) and the Many Labs projects (Klein et al., 2014, 2018). In addition to being the first large-scale replication projects in psychology, they also illustrate the different approaches that can be taken to multi-site replication work. The OSC project is an example of a “broad-and-shallow” approach to direct replications, in which single replications of many different findings were carried out, each at a different site. The OSC conducted replications of 100 experimental and correlational studies from cognitive and social psychology. They reported that effect sizes were approximately half the magnitude of the original effects, and only 37% of replications showed significant results (Open Science Collaboration, 2015). In contrast, the two Many Labs projects are an example of a “narrow-and-deep” approach, in which the authors seek to replicate the same small group of findings across a number of sites with some variation, mainly in testing population. The findings varied across the two Many Labs projects, but in both cases the authors concluded that replicability depended more upon the effect being studied than the sample or setting used to study it (Ebersole et al., 2016; Klein et al., 2018). Besides these two recent efforts, it is also worth noting that the idea of multi-site replication is now starting to become embedded in undergraduate education, for example via the establishment of the GW4 Undergraduate Psychology Consortium in the United Kingdom (Button et al., 2019).

As described above, we define *direct replication* studies as those which aim to keep the material realization of the primary information focus as close to the original study as possible and c*onceptual replications* as those, which test the same purported mechanisms with different materials. Both the Reproducibility Project and the Many Labs projects are direct replication studies, which closely reproduce the material realization while varying the contextual background. Indeed, the replication protocols were developed whenever possible in collaboration with the original authors, even including the use of original materials. However, direct replications are not a panacea. Among other things, simply reproducing methodologies without considering theoretical underpinnings [what Phaf (2020) calls “mechanical” replications] runs the risk of perpetuating, rather than unearthing, problems. As Gelman and Carlin (2014) explain, “Consistent findings could take on the status of confirmed truths, when they actually reflect failings in study design, methods or analytical tools.” (p400).

A number of suggestions have been advanced to improve the quality of replications. Phaf (2020) suggested that experimental work should always be complemented by thorough theoretical analyses. In the case of unsuccessful replications, this would allow for the discovery of potentially crucial (and as yet unexamined) factors that may explain the result. A second related suggestion is to formulate competing theoretical hypotheses that focus on the disproof and exclusion of alternative explanations rather than the traditional presence or absence of a statistical effect (for detailed discussions see Platt, 1964; Phaf, 2020). Adopting this approach in the field of cognitive hearing science would minimize the existence of null results and replication failures. Such a change in hypothesis generation would, however, necessitate development of and closer engagement with underlying theoretical concepts. A third approach focuses on paying closer attention to the types of errors that occur as part of incorrect statistical inferences and effect size estimation. Two types of errors are often differentiated: errors of magnitude (in which the effect size is exaggerated) and errors of direction. Both types of errors can be surprisingly high for underpowered studies, even when the statistical results are significant (Gelman and Carlin, 2014). In the context of our discussion, this means that if studies are underpowered, their results may not only be non-significant (thereby leading to replication failure) but may also give rise to effects in the unexpected direction. In a study with theoretically-motivated hypotheses, such a misdirected effect would likely be discounted regardless of its significance. However, in a study that only predicts an effect of a variable without specifying its direction (common in regression-type analyses of individual differences), it is much harder to identify errors of direction. For a detailed discussion of the probabilities for these types of errors see Kirby and Sonderegger (2018). Finally, some researchers advocate the adoption of “big data” and machine learning to enhance reproducibility in psychological research – approaches which, among other things, involve very large sample sizes (Yarkoni and Westfall, 2017). This may, the authors suggest, involve using existing datasets or corpora, or it may involve “large, multilab, collaborative projects” (p. 1110), such as the OSC and Many Labs projects – a point to which we return below. Of course, not all researchers will wish or be able to involve machine learning in their work; however, regardless of whether or not one takes an AI-based approach, it is clear that increased sample sizes are vital in order to avoid errors resulting from underpowering or overfitting to local noise. Similarly, clearly defined and pre-determined stopping rules for data collection must be implemented to reduce the prevalence of false-positive results (Simmons et al., 2011). Recent developments in the area of stopping rules have shown them to have important implications in both frequentist and Bayesian hypothesis testing (Rouder, 2014; Sanborn and Hills, 2014).

Conceptual replications, meanwhile, have also been subject to criticism. For example, Pashler and Harris (2012) argue that results from direct replications always have the power to advance the field: successes strengthen the trust in the phenomenon, while failures will slowly erode it. However, while successful conceptual replications provide new information by extending the reach of the phenomenon, failures of conceptual replications will not necessarily erode the trust in a phenomenon and thus not provide useful information. Failures will only be interpreted as showing that the material realization was not close enough to the original study. Such an interpretation cannot exclude the possibility that the phenomenon itself (with the same material realization) may not have been replicable in the first place. In this sense, conceptual replications may have less information value than direct replications. Arguing along similar lines, Nosek and Errington (2020) suggest that many conceptual replications are in practice actually generalizability tests, in which failures “are interpreted, at most, as identifying boundary conditions” (p. 5).

Nevertheless, we argue that – regardless of whether they are viewed as “replications” proper or as generalizability tests – conceptual replications have both practical and theoretical value. From a practical perspective, many attempts at replication are conceptual to some extent: materials and methods are often based only on the descriptions given in the experimental report, and these are typically underspecified (Open Science Collaboration, 2015). It is therefore important to determine whether the level of change in materials used in a replication study mean that it is a “meaningful” conceptual replication (if successful), or whether the changes are simply unavoidable but non-critical variability in the material realization of the primary information focus, thus making the study effectively a direct replication. Such conceptual replications are vital if researchers want to know which particular implementation of a given task is likely to produce the most robust effect in their participant pool, and/or which specific details of a set-up are vital and which can be safely varied or omitted.

From a theoretical perspective, conceptual replications are important because they can add further support to the original hypotheses and/or proposed mechanisms underlying a particular effect; indeed, by identifying boundary conditions in terms of experimental protocol, they can actually help to clarify and refine the original interpretation and explanation of an effect. In order for conceptual replications to provide all of this information, they need to be carried out in a systematic and incremental fashion, altering only one aspect of a single class of variable at a time. Unfortunately, as Schmidt (2009) observes, conceptual replications are relatively unpopular with reviewers and editors, and as a result, the process of conceptual replication is often not explicit – and therefore somewhat haphazard.

However, conceptual replications do present a theoretical complication. A pure conceptual replication should vary the material realization of the primary information focus, not the contextual background; therefore, strictly speaking, they should be carried out using an identical participant sample to the original study (Schmidt, 2009). In reality, of course, this is not possible or practical. In order to carry out conceptual replications in as meaningful a way as possible, one should therefore perform them over a large enough sample and variety of sites to demonstrate *both* robustness of the concept itself *and* its replicability over multiple contexts. Such a large-scale study would both (i) function as a conceptual replication that explores in a controlled and systematic fashion the necessary and sufficient material conditions required for an effect to emerge and reveals meaningful boundary conditions and also (ii) use large enough sample sizes to be able to discount sampling error, artifacts and lack of power as explanations for the effects. One way to address these issues is to run a series of systematic multi-lab conceptual replications [along the lines of the large, collaborative projects advocated by Yarkoni and Westfall (2017) see above]. In the following section, we present one example of how such an approach might work in practice, focusing on a test commonly used to assess inhibition – the Stroop task.

## Direct and Conceptual Replications of Group Effects and Individual Differences in the Context of Stroop Tasks

Stroop tasks are widely used to assess inhibition – the ability to suppress goal-irrelevant information (Stroop, 1935; MacLeod, 1991). In its classic form, the Stroop task assesses inhibition in the visual domain via color-word interference. Participants are required to name the ink color of a string of characters while ignoring the characters themselves. In the neutral condition, these characters are meaningless or irrelevant; in the incongruent condition, they spell out a conflicting color word (e.g., BLUE printed in red). The difference in reaction times between the incongruent and neutral conditions is typically taken as a measure of inhibitory ability and termed Stroop interference (SI).

The visual Stroop task is an example of a task with a rich conceptual replication history, particularly as concerns the testing materials. For example, some studies enhance the visibility of the color by replacing font color with a larger patch of color underneath a superimposed word (Janse, 2012; Knight and Heinrich, 2017). For the control condition, some studies use a string of Xs as their irrelevant characters, while others use unrelated words or even simply blank patches of color (MacLeod, 1991). In the incongruent condition, meanwhile, some studies have used only the first letters of the incongruent color words (such as “R” instead of “RED”; Regan, 1978). Such conceptual replications have been shown to vary the size of the interference effect, but as a general rule such modifications “only modestly affect its magnitude, not its qualitative form” (MacLeod, 1991, p166). Indeed, even with substantial changes to experimental protocol, Stroop-type tasks still produce an interference effect; such changes include – to name just a few – spatial separation of color patches and words, using different response modalities (oral vs. manual), using color-related (as opposed to actual color) words in the incongruent condition (e.g., lemon and sky), and asking participants to sort stimuli into categories rather than simply naming or otherwise responding to their basic properties.

This rich and robust replication history stands in contrast to auditory versions of the Stroop task. Although such versions have been successfully used (Green and Barber, 1981; Morgan and Brandt, 1989), their replication appears to be less successful if we take as an indication the rarity of published studies reporting them. In auditory Stroop tasks, participants are typically required to respond to some perceptual feature of a sound while ignoring the semantic content, which – as in the visual version – can be either irrelevant or conflicting. For example, participants may be required to respond to the speaker’s gender regardless of the word spoken, which in the control condition will be neutral (e.g., “cat”) and in the incongruent condition will be conflicting (e.g., “woman” spoken by a man). In addition to gender, other auditory dimensions have been used including pitch (“high” vs. “low”), location (“left” vs. “right”), loudness (“loud” vs. “soft”), and even time (“fast” vs. “slow”) (Hamers and Lambert, 1972; Pieters, 1981; Morgan and Brandt, 1989; Roberts and Hall, 2008; Whitton et al., 2017). As well as fewer studies reporting the use of the task, there are also direct reports of non-replication. For example, Morgan and Brandt (1989) report an auditory Stroop interference effect only in the pitch domain, but not in the time domain. Additionally, Knight and Heinrich (2017) found a modest auditory Stroop interference effect using a gender-based task only on the group level, but could not replicate this effect for every participant or indeed for every one of the four speakers used in their materials.

Auditory versions of the Stroop task are particularly attractive when the main outcome variable of interest is itself auditory – for example, speech-in-noise perception – and have been used both alone and alongside visual Stroop tasks (Sommers and Danielson, 1999; Knight and Heinrich, 2017; Whitton et al., 2017). In many cases, it is implied that the auditory Stroop task is essentially equivalent to a visual version: for example, immediately beneath the heading “Audio/Visual Stroop,” Whitton et al. (2017) simply state that “The Stroop effect provides a well-established measure of inhibitory control.” Here, the auditory Stroop task is being treated as a conceptual equivalent of the visual Stroop: a task which, despite the very different material realization of the primary information focus, nevertheless produces the same group-level effects (and presumably therefore taps into the same underlying mechanism) as the classic visual version.

However, in the case of the auditory Stroop task, this is in fact far from clear. In 1991, MacLeod asked “How equivalent are all of these tasks that superficially resemble the Stroop task? Even for the very prevalent alternatives […] we do not know […] Obviously, though, it is of theoretical importance to know whether similar processes are invoked in these many variations, but we have insufficient evidence at present.” (MacLeod, 1991, p. 170). This remains true for the auditory Stroop task nearly 30 years later: although it is often assumed to tap the same underlying domain-general inhibitory ability as the visual task, the extent to which this is true is unclear. Crucially, the extent of overlap appears to depend on the exact implementation of the two tasks. For example, Roberts and Hall (2008), using extremely carefully chosen and closely matched tasks, demonstrated similar patterns of neural activation and correlated behavioral responses for Stroop tasks presented across different modalities, suggesting that visual and auditory versions do indeed tap shared inhibitory processes. Conversely, when auditory and visual Stroop tasks were less closely matched and arguably more *conceptually* similar than methodologically similar, the auditory and visual versions have not been found to correlate at all between individuals (Shilling et al., 2002; Knight and Heinrich, 2017).

In short, then, there are a number of reproducibility issues regarding the auditory Stroop task that need to be addressed. First, at the level of group effects, more conceptual replications are needed of the auditory Stroop task in isolation in order to investigate which specific material realizations (e.g., gender- vs. pitch-based tasks) produce a reliable group-level effect in the auditory domain. Second, at the level of individual differences, direct replications of individual scores (i.e., retest reliability) need to be considered. We are not aware of any studies that have provided these data for auditory Stroop tasks, and not having this information limits our understanding of the extent to which correlations between different Stroop tasks are limited by retest reliability (Hedge et al., 2018). Finally, even if measures of auditory Stroop interference are replicable across different material realizations and reliably assess behavior on an individual basis, the question remains of whether or not they assess the same underlying mechanism as the visual Stroop task. Therefore, research needs to assess whether participants’ individual scores are correlated (i.e., replicate) across the two types of task in their different material realizations. Only if this is true can the visual and auditory Stroop tasks be considered *conceptually* equivalent.

Besides these theoretical considerations, there is also a strong practical aspect to such a project: if researchers know that auditory Stroop tasks do (or do not) produce similar results to their visual counterparts and are aware of which auditory Stroop implementations produce the largest and/or most visual-like results, then they can confidently select the best type of Stroop task for their purposes. We believe that these questions could be fruitfully addressed using a many-labs-style replication project and outline in the following section how such a project could be implemented.

## Setting Up a Multi-Lab Replication for the Auditory Stroop

Any number of implementations of the auditory Stroop task could be tested, but in the first instance it seems reasonable to attempt to replicate a small number of tasks already reported by existing studies. Such a practice would also be consistent with the traditional approach to replication: that is, selecting previously reported key results and seeking to replicate their group effects as closely as possible. In addition to the selected auditory Stroop tasks, we would suggest the inclusion of a classic color-word visual Stroop, since this is in many ways the “gold standard” version of the task (MacLeod, 1992). In terms of participants, a conceptual replication would imply the use of a sample as closely matched demographically to the original studies as possible – in this case, undergraduate students. However, a straightforward extension of the replication could see the demographic requirements for participation relaxed and the influence of demographic variables on the Stroop effect investigated in its own right. In this case, it would be necessary to collect demographic information about all participants, along with measures of visual and auditory acuity.

The selected auditory Stroop tasks, the color-word visual Stroop task and the collection of relevant demographic data would serve as a core package carried out across all sites involved in the study. Following the Hendrick (1991) and Schmidt (2009) framework, we suggest that this core package keeps the immaterial realization of the primary information focus constant while varying its material realization – in other words, it provides a conceptual replication by testing the robustness and replicability of results across different Stroop tasks. Rolling out this core package across multiple labs also varies critical aspects of the contextual background, testing replicability across different participant groups, physical settings and experimenters. This approach therefore fulfills the need, outlined above, for conducting conceptual replications over a large enough sample and variety of sites to demonstrate *both* robustness of the concept itself *and* its replicability over multiple contexts. It is nevertheless desirable to minimize those aspects of the contextual background that Schmidt refers to as *specific task variables*: minor variations in materials such as paper color, headphone type, screen resolution and so forth. To minimize these effects in our core package, we suggest using the same stimuli across all labs involved in the project, and using shared calibration procedures and close collaboration during task set-up. Online repositories for sharing materials – such as that hosted by the Open Science Framework^2^ –are of great help in this regard: participating labs can easily and remotely access not only stimuli, but also details of calibration procedures and code for running the tasks, thus ensuring that set-up, instructions, and procedure are as close as possible across the different participating sites.

Besides the closely prescribed core package, which would be relatively brief, participating laboratories could also have flexibility in adding their own tasks and collecting supplementary, single-lab datasets relevant to their needs and interests. One key addition, as discussed above, would be to ask participants to perform the same tasks multiple times to assess retest reliability. Individual labs may also be interested in running the tasks on different listener groups or adding additional tasks to explore the relationship of Stroop scores to other measures. A further extension could be a comparison of data collected online with that collected in the laboratory. The growing popularity of online recruitment and/or testing platforms such as Gorilla^3^, Prolific^4^, and Amazon’s Mechanical Turk^5^ has opened up possibilities for collecting data from a much broader range of participants than those typically involved in laboratory studies^6^. Another line of extensions could explore the limits of replicability by changing aspects of the set-up in ways that theoretical analyses suggest alter the task in a conceptually meaningful manner. Such replications (or replication failures) would help delineate the extent to which generalizations can reasonably be made [see the suggestions of Phaf (2020) and others, discussed above].

## Conclusion

In this article, we have discussed theoretical and practical aspects of the reproducibility crisis in science and how they might be tackled. In particular, we have suggested that one way to improve reproducibility, particularly when assessing individual differences, is to encourage researchers to include retest reliability measures of their quantitative assessment methods as a routine aspect of testing, analysis, and reporting. The gradual collection of retest coefficients of commonly-used tests in a variety of situations would allow researchers to better judge the reliability of tests, which in turn should influence both the planning stage of studies as well as the interpretation of results. We have also advocated for both direct replications – those which address the contextual background of a task while preserving the material realization of the primary information focus as far as possible – and also conceptual replications. In particular, we have focused on the benefits of large-scale systematic conceptual replications – that is, systematically varying the material realization of the primary information focus while nevertheless collecting large enough, multi-site sample sizes to account for contextual variation. Such replications can only be achieved through close collaboration on multi-lab projects.

## Author Contributions

AH and SK wrote the manuscript with oversight and conceptual guidance from AH. AH also produced the final structure of the article. Both authors contributed to the article and approved the submitted version.

## Conflict of Interest

The authors declare that the research was conducted in the absence of any commercial or financial relationships that could be construed as a potential conflict of interest.

## Footnotes

1 We only include here cognitive tests that have previously been shown to have a link with speech perception and/or communication.

2 https://osf.io/

3 www.gorilla.sc

4 www.prolific.ac

5 www.mturk.com

6 Early indications are that data quality and reliability is high for online studies (Casler et al., 2013; Gould et al., 2015) but disparities between lab and online samples do emerge for some tasks (Crump et al., 2013). As a result, a new facet of replicability has been added to the contextual background of a study: how well results replicate across lab and online cohorts. Both visual and auditory Stroop tasks can be set up in such a way that the same task can be run online and in the lab, thus allowing this additional aspect of reproducibility to be assessed.
