# Supplementary material for: Reproducibility in Cognitive Hearing Research: Theoretical Considerations and Their Practical Application in Multi-Lab Studies
Source: Front Psychol. 2020 Jul 16;11:1590. doi: 10.3389/fpsyg.2020.01590 (PMC7378399; doi:10.3389/fpsyg.2020.01590)
Supplement: Supplementary file 1 [file Data_Sheet_1.pdf]

Supplementary Material

Supplementary Table 1

Supplementary Table 1. Examples of studies providing retest reliability coefficients in three areas of research: 1) cognition, 2) speech perception and 3) self-reported measures of communication. HL = hearing loss; MMHL = mild-to-moderate hearing loss; HA = hearing aid users; NH = normal hearing; ICC = Intraclass Correlation Coefficient, PPMC = Pearson's Product-Moment Correlation; ? = information not provided

**1 Cognition**								
**Test**	**Sample Size (N)**	**Age range**	**Group tested**	**Time between administrations (mean/median)**	**Type of test-retest coefficient**	**Value**	**Systematic difference between sessions (e.g., Practice / memory effects)**	**Reference**
Listening Span	42	19 – 35<br>24.4 (mean)	NH	2 – 4 weeks	ICC	0.66	Yes	Besser, Koelewijn, Zekveld, Kramer, and Festen (2013)
Reading Span						0.69		
Digit Span (forward & backward)	21	50 – 74<br>65.0 (mean)	Mild HL	4 weeks	ICC	0.88	No	Ferguson and Henshaw (2015)<br>Ferguson, Henshaw, Clark, and Moore (2014) for information on demography, design, practice effects
Visual letter monitoring task - slow						0.70		
Visual letter monitoring task - fast						0.72		
TEA single (T6)						0.93		
TEA dual (T7)						0.83		
TEA dual task decrement						0.72		
Dual Task digit recall – Quiet	30	50 - 74<br>67.4 (mean)	MMHL, HA users	1 week	ICC	0.81	?	Ferguson and Henshaw (2015)<br>Henshaw and Ferguson (2014) for information on demography, design, practice effects
Dual Task digit recall – 0 dB						0.76		
Dual Task digit recall – -4 dB						0.72		
Letter Number Sequencing						0.83		

Test	Sample Size (N)	Age range	Group tested	Time between administrations (mean/median)	Type of test-retest coefficient	Value	Systematic difference between sessions (e.g., Practice / memory effects)	Reference
Text Reception Threshold	13	53 – 78 63.5 (mean)	NH	minutes	Spearman-Brown	0.88	?	George et al. (2007)
	21	46 – 81 65.5 (mean)	MMHL			0.87		
Recognition memory (RMT) - words	56	40 – 54	Healthy	1 month	PPMC	0.33 (non-identical version)	No	Bird, Papadopoulou, Ricciardelli, Rossor, and Cipolotti (2003)
	56	40 – 54				0.66 (identical version)	No	
	56	55 – 70				0.57 (non-identical version)	No	
	56	55 – 70				0.76 (identical version)	Yes	
Digit Symbol Test	188	39 - 75	Healthy	1 month	PPMC / Spearman	0.82	Yes	Bird, Papadopoulou, Ricciardelli, Rossor, and Cipolotti (2004)
Phonological fluency	99					0.63		
Semantic fluency	99					0.56		
Modified Wisconsin Card Sorting Test	90					0.16 – 0.38		
Logical memory immediate	25	50. 1 (mean)	Hypertensives	7 – 10 days	PPMC	0.61	Yes	McCaffrey, Ortega, Orsillo, Nelles, and Haase (1992)
Logical memory delayed	25					0.74	Yes	
Figural memory immediate	24					0.63	Yes	
Figural memory delayed	24					0.74	Yes	
Paired associates	25					0.53	Yes	
Trail Making A	25					0.80	No	
Trail Making B	24					0.92	No	
Attention Span	24					0.78	No	
Digit Span forward	24					0.78	No	
Digit Span backward	24					0.85	Yes	
Logical memory immediate	33	59.1 (mean)	Chronic cigarette smokers			0.47	Yes	
Logical memory delayed	32					0.68	Yes	
Figural memory immediate	32					0.53	Yes	
Figural memory delayed	32					0.69	Yes	
Trail Making B	32					0.49	Yes	
Phonological fluency 3 letters (FAS)	90	Working age	Healthy	1 – 8 weeks	PPMC	0.82	?	Harrison, Buxton, Husain, and Wise (2000)
Phonological fluency letter B	90					0.73		
Semantic fluency	90					0.68		

Test	Sample Size (N)	Age range	Group tested	Time between administrations (mean/median)	Type of test-retest coefficient	Value	Systematic difference between sessions (e.g., Practice / memory effects)	Reference
Verbal IQ	122	57 – 85<br>70.4 (mean)	Healthy	3 administrations, each 1 year apart	?	0.80 (Y1-Y2)<br>0.82 (Y2-Y3)	?	Mitrushina and Satz (1991)
Performance IQ						0.70 (Y1-Y2)<br>0.58 (Y2-Y3)		
Full Scale IQ						0.83 (Y1-Y2)<br>0.79 (Y2-Y3)		
Digit Span						0.64 (Y1-Y2)<br>0.64 (Y2-Y3)		
Trail Making A						0.53 (Y1-Y2)<br>0.56 (Y2-Y3)		
Trail Making B						0.67 (Y1-Y2)<br>0.67 (Y2-Y3)		
Immediate Recall						0.68 (Y1-Y2)<br>0.81 (Y2-Y3)		
Delayed Recall						0.62 (Y1-Y2)<br>0.79 (Y2-Y3)		
Rey Auditory Verbal Learning test						0.41 (Y1-Y2)<br>0.79 (Y2-Y3)		
Visual Reproduction immediate						0.68 (Y1-Y2)<br>0.71 (Y2-Y3)		
Visual Reproduction delayed						0.64 (Y1-Y2)<br>0.68 (Y2-Y3)		
Complex Figure immediate						0.56 (Y1-Y2)<br>0.58 (Y2-Y3)		
Complex Figure delayed						0.57 (Y1-Y2)<br>0.77 (Y2-Y3)		
Boston Naming Test						0.62 (Y1-Y2)<br>0.65 (Y2-Y3)		
Gibson Test Long-term memory	21	18 - 58	Healthy	2 weeks	PPMC	0.67	?	Moore and Miller (2018)
Gibson Test Short-term memory						0.75		
Gibson Test Processing speed						0.76		
Gibson Test Word attack						0.68		
Gibson Test Visual Processing						0.74		
Gibson Test Auditory Processing						0.77		
Gibson Test Logic & Reasoning						0.66		
Simon Stop-Signal Task, GO trials RT	16	18 – 34<br>24 (mean)	Healthy	3 administrations, 1 week apart	PPMC / Spearman	0.53 (W1-W2)<br>0.64 (W2-W3)	Yes<br>No	Palmer, Langbehn, Tabrizi, and Papoutsi (2018)
Simon Stop-Signal Task, SS interference effect RT						-0.08 (W1-W2)<br>0.11 (W2-W3)	No<br>No	
Trail Making Test A						0.52 (W1-W2)<br>0.55 (W2-W3)	Possibly	
Trail Making Test B						0.76 (W1-W2)<br>0.66 (W2-W3)	No<br>No	

Test	Sample Size (N)	Age range	Group tested	Time between administrations (mean/median)	Type of test-retest coefficient	Value	Systematic difference between sessions (e.g., Practice / memory effects)	Reference
Category naming	15	23.9 (mean)	Healthy	10-12 days	ICC	0.78	No	Vora, Varghese, Weisenbach, and Bhatt (2016)
Word list generation						0.78		
Letter Number Sequencing						0.65		
Colour naming (Stroop)						0.78 (Congruent time)		
						0.92 (Incongruent time)		
Triangle and letter tracking						0.71 (Triangle)		
						0.72 (Letter)		
Spot and click						0.84 (Simple RT)		
						0.89 (Choice RT)		
Unveil the star						0.77 (Total time)		
						0.95 (Error)		
Peg board game						0.91 (Total time)		
						0.89 (Error)		
Simon task	30	65 – 71<br>68.1 (mean)	Healthy	3 sessions, each 1 week apart; 2 administrations within a session	ICC	0.54 (W1-W2, RT Compatible)<br>0.69 (W2-W3, RT Compatible)	No	White, Flannery, McClintock, and Machado (2019) (coefficients for first administrations within each session, i.e. T1, T3, T5)
						0.63 (W1-W2, RT Incompatible)<br>0.73 (W2-W3, RT Incompatible)	No	
						0.57 (W1-W2, RT Cost)<br>0.29 (W2-W3, RT Cost)	No	
Flanker task						0.56 (W1-W2, RT Compatible)<br>0.76 (W2-W3, RT Compatible)	Yes	
						0.51 (W1-W2, RT Incompatible)<br>0.69 (W2-W3, RT Incompatible)	Yes	
						0.11 (W1-W2, RT Cost)<br>0.41 (W2-W3, RT Cost)	No	
Corsi Span forward						0.32 (W1-W2, Span)<br>0.17 (W2-W3, Span)	Yes	
						0.41 (W1-W2, Scores)<br>0.33 (W2-W3, Scores)	Yes	
Corsi Span backward						0.39 (W1-W2, Span)<br>0.21 (W2-W3, Span)	Possibly	
						0.35 (W1-W2, Score)<br>0.29 (W2-W3, Score)	Possibly	
2-back task						0.33 (W1-W2, Accuracy)<br>0.73 (W2-W3, Accuracy)	No	
						0.56 (W1-W2, RT)<br>0.78 (W2-W3, RT)	Yes	

2 Speech tests								
**Test**	**Sample Size (N)**	**Age range**	**Group tested**	**Time between administrations (mean/median)**	**Type of test-retest coefficient**	**Value**	**Systematic difference between sessions (e.g., Practice / memory effects)**	**Reference**
NU6 word lists in quiet	10	30 - 81	MMHL	5 weeks	correlation, not further specified	0.92 – 0.96	Yes (?)	Causey, Hermanson, Hood, and Bowling (1983)
NU6 word lists in competing message at -8 dB SNR	24	17 - 35<br>23 (mean)	NH	?	PPMC	0.9	Yes	Stoppenbach, Craig, Wiley, and Wilson (1999)
NU6 word lists in competing message at +4 dB SNR						0.8		
Digit Triplet Test (words) in 8-Hz modulated noise	21	50 – 74<br>65.0 (mean)	Mild HL	4 weeks	ICC	0.87	No	Ferguson and Henshaw (2015)<br>Ferguson et al. (2014) for information on demography, design, practice effects
ASL sentence test in speech-shaped noise						0.67		
Dual task word repetition - Quiet	30	50 – 74<br>67.4 (mean)	MMHL, HA users	1 week	ICC	0.90	?	Ferguson and Henshaw (2015)<br>Henshaw and Ferguson (2014) for information on demography and design
Dual task word repetition – 0 dB						0.80		
Dual task word repetition - -4 dB						0.35		
Modified coordinate response measure						0.45		
IEEEE sentences in 4-talker babble	20	?	NH	1 – 14 days	correlation, not further specified	0.80 (composite score)<br>0.92 (all contributing scores kept separate)	?	Bentler (2000)
	20	?	MMHL	1 – 14 days	correlation, not further specified	0.92 (composite score)<br>0.97(all contributing scores kept separate)	?	

Test	Sample Size (N)	Age range	Group tested	Time between administrations (mean/median)	Type of test-retest coefficient	Value	Systematic difference between sessions<br>(e.g., Practice / memory effects)	Reference
SRT of sentences in stationary noise	13	53 – 78<br>63.5 (mean)	NH	minutes	Spearman-Brown	0.60	?	George et al. (2007)
SRT of sentences in modulated noise						0.88		
SRT of sentences in stationary noise	21	46 – 81<br>65.5 (mean)	MMHL	minutes	Spearman-Brown	0.83		
SRT of sentences in modulated noise						0.96		
SRT of words in 6-talker babble	315	69.7 (mean)	Mild-to-severe HL	12 months	ICC	0.88	Yes	Wilson and McArdle (2007)
SRT of words in 6-talker babble	48	54 – 86<br>66.9 (mean)	Mild HL	14 – 89 days<br>39.5 days (mean)	ICC	0.89	No	
SRT of words in 6-talker babble	48	52 – 87<br>71.9 (mean)	Moderate HL	21 – 130 days<br>42.1 days (mean)	ICC	0.91	No	

3 Speech communication self-report measures (questionnaires)								
**Test**	**Sample Size (N)**	**Age range**	**Group tested**	**Time between administrations (mean/median)**	**Type of test-retest coefficient**	**Value**	**Systematic difference between sessions (e.g., Practice / memory effects)**	**Reference**
COSI	98	71 (mean)	MMHL HA users	16 weeks	PPMC	0.73 (Improvement)<br>0.84 (Final ability)	?	Dillon, James, and Ginis (1997)
GHABP	293 (?)		MMHL HA users	3 weeks	PPMC	0.86	?	Gatehouse (1999)
HERE	50	61 – 86<br>69 (mean)	Mild HL	158 days (mean)	ICC	0.86 (total)<br>0.84 (speech)<br>0.82 (spatial)<br>0.80 (social)	No	Heinrich, Mikkola, Polku, Törmäkangas, and Viljanen (2019)
HHIA	28	28 – 59<br>44.2 (mean)	NH to Mild HL	6 weeks	PPMC	Face – Written:<br>0.97 (total score)<br>0.93 (emotional)<br>0.95 (social/situational)	No	Newman, Weinstein, Jacobson, and Hug (1991)
HHIA -S						Face – Written:<br>0.93 (total score)<br>0.88 (emotional)<br>0.82 social/situational)	No	
HHIE	20	61-92<br>80 (mean)	MMHL	6 weeks	PPMC	Face-to-face:<br>0.96 (total)<br>0.98 (emotional)<br>0.92 (social)	No	Weinstein, Spitzer, and Ventry (1986)
	27					Written:<br>0.84 (total)<br>0.79 (emotional)<br>0,.80 (social)		
	98	71 (mean)	MMHL HA users	6 weeks	PPMC	0.57 (total)<br>0.54 (emotional)<br>0.58 (social)	?	Dillon et al. (1997)
Localisation Disabilities and Handicaps Q	20	25 – 78<br>49.65 (mean)	Severe-to-profound unilateral HL	3 weeks	PPMC	Disabilities: 0.90<br>Handicaps: 0.70	?	Ruscetta, Palmer, Durrant, Grayhack, and Ryan (2005)
	10	23 – 73<br>48 (mean)	Bilateral MMHL					

Test	Sample Size (N)	Age range	Group tested	Time between administrations (mean/median)	Type of test-retest coefficient	Value	Systematic difference between sessions (e.g., Practice / memory effects)	Reference
SSQ	159	60 - 88	Very mild HL	6 months	PPMC			Singh and Pichora-Fuller (2010)
	40	72.4 (mean)				interview – interview<br>0.83 (total)<br>0.77 (speech)<br>0.86 (spatial)<br>0.83 (qualities)	?	
	39	71.6 (mean)				interview – mail<br>0.66 (total)<br>0.71 (speech)<br>0.49 (spatial)<br>0.65 (qualities)		
	40	73.2 (mean)				mail – interview<br>0.69 (total)<br>0.74 (speech)<br>0.59 (spatial)<br>0.69 (qualities)		
	40	73.8 (mean)				mail – mail<br>0.65 (total)<br>0.83 (speech)<br>0.56 (spatial)<br>0.64 (qualities)		

References

Bentler, R. A. (2000). List equivalency and test-retest reliability of the speech in noise test. *American Journal of Audiology, 9*(2), 84-100.

Besser, J., Koelewijn, T., Zekveld, A. A., Kramer, S. E., & Festen, J. M. (2013). How linguistic closure and verbal working memory relate to speech recognition in noise - a review. *Trends in Amplification, 17*(2), 75-93. doi:10.1177/1084713813495459

Bird, C. M., Papadopoulou, K., Ricciardelli, P., Rossor, M. N., & Cipolotti, L. (2003). Test–retest reliability, practice effects and reliable change indices for the recognition memory test. *British Journal of Clinical Psychology, 42*, 407–425. doi:10.1348/014466503322528946

Bird, C. M., Papadopoulou, K., Ricciardelli, P., Rossor, M. N., & Cipolotti, L. (2004). Monitoring cognitive changes: Psychometric properties of six cognitive tests. *British Journal of Clinical Psychology, 43*, 197-210.

Causey, G. D., Hermanson, C. L., Hood, L. J., & Bowling, L. S. (1983). A comparative evaluation of the Maryland NU 6 auditory test. *Journal of Speech and Hearing Disorders, 48*, 62-69.

Dillon, H., James, A., & Ginis, J. (1997). Client Oriented Scale of Improvement (COSI) and its relationship to several other measures of benefit and satisfaction provided by hearing aids. *Journal of the American Academy of Audiology, 8*(1), 27-43.

Ferguson, M. A., & Henshaw, H. (2015). How does auditory training work? Joined-p thinking and listening. *Seminars in Hearing, 36*(4), 237-249.

Ferguson, M. A., Henshaw, H., Clark, D. P., & Moore, D. R. (2014). Benefits of phoneme discrimination training in a randomized controlled trial of 50- to 74-year-olds with mild hearing loss. *Ear and Hearing, 35*(4), e110-121. doi:10.1097/AUD.0000000000000020

Gatehouse, S. (1999). Glasgow Hearing Aid Benefit Profile: derivation and validation of a client-centered outcome measure for hearing-aid services. *Journal of the American Academy of Audiology, 10*, 80-103.

George, E. L., Zekveld, A. A., Kramer, S. E., Goverts, S. T., Festen, J. M., & Houtgast, T. (2007). Auditory and nonauditory factors affecting speech reception in noise by older listeners. *Journal of the Acoustical Society of America, 121*(4), 2362-2375. doi:10.1121/1.2642072

Harrison, J. E., Buxton, P., Husain, M., & Wise, R. (2000). Short test of semantic and phonological fluency: Normal performance, validity and test-retest reliability. *British Journal of Clinical Psychology, 39*, 181-191.

Heinrich, A., Mikkola, T. M., Polku, H., Törmäkangas, T., & Viljanen, A. (2019). Hearing in Real-Life Environments (HERE): Structure and reliability of a questionnaire on perceived hearing for older adults. *Ear and Hearing, 40*(2), 368-380. doi:10.1097/AUD.0000000000000622

Henshaw, H., & Ferguson, M. A. (2014). *Assessing the benefits of auditory training to real-world listening: Identifying appropriate and sensitive outcomes.* Paper presented at the ISAAR 2013: Auditory Plasticity—Listening with the Brain. 4th Symposium on Auditory and Audiological Research, Nyborg, Denmark.

McCaffrey, R. J., Ortega, A., Orsillo, S. M., Nelles, W. B., & Haase, R. F. (1992). Practice effects in repeated neuropsychological assessments. *The Clinical Neuropsychologist, 6*(1), 32-42. doi:10.1080/13854049208404115

Mitrushina, M., & Satz, P. (1991). Effect of repeated administration of a neuropsychological battery in the elderly. *Journal of Clinical Psychology, 47*(6), 790–801.

Moore, A. L., & Miller, T. M. (2018). Reliability and validity of the revised Gibson Test of Cognitive Skills, a computer-based test battery for assessing cognition across the lifespan. *Psychology Research and Behavior Management, 12*(11), 25-35. doi:10.2147/PRBM.S152781

Newman, C. W., Weinstein, B. E., Jacobson, G. P., & Hug, G. A. (1991). Test-retest reliability of the hearing handicap inventory for adults. *Ear and Hearing, 12*(5), 355-357.

Palmer, C. E., Langbehn, D., Tabrizi, S. J., & Papoutsi, M. (2018). Test-retest reliability of measures commonly used to measure striatal dysfunction across multiple testing sessions: A longitudinal study. *Frontiers in Psychology, 8*, 2363. doi:10.3389/fpsyg.2017.02363

Ruscetta, M. N., Palmer, C. V., Durrant, J. D., Grayhack, J., & Ryan, C. (2005). Validity, internal consistency, and test/retest reliability of a localization disabilities and handicaps questionnaire. *Journal of the American Academy of Audiology, 16*(8), 585-595.

Singh, G., & Pichora-Fuller, K. M. (2010). Older adults performance on the speech, spatial, and qualities of hearing scale (SSQ): Test-retest reliability and a comparison of interview and self-administration methods. *International Journal of Audiology, 49*(10), 733-740. doi:10.3109/14992027.2010.491097

Stoppenbach, D. T., Craig, J. M., Wiley, T. L., & Wilson, R. H. (1999). Word recognition performance for Northwestern University Auditory Test No. 6 word lists in quiet and in competing message. *Journal of the American Academy of Audiology, 10*(8), 429-435.

Vora, J. P., Varghese, R., Weisenbach, S. L., & Bhatt, T. (2016). Test-retest reliability and validity of a custom-designed computerized neuropsychological cognitive test battery in young healthy adults. *Journal of Psychology and Cognition, 1*(1), 11-19.

Weinstein, B. E., Spitzer, J. B., & Ventry, I. M. (1986). Test-retest reliability of the Hearing Handicap Inventory for the Elderly. *Ear and Hearing, 7*(5), 295-299.

White, N., Flannery, L., McClintock, A., & Machado, L. (2019). Repeated computerized cognitive testing: Performance shifts and test-retest reliability in healthy older adults. *Journal of Clinical and Experimental Neuropsychology, 41*(2), 179-191. doi:10.1080/13803395.2018.1526888

Wilson, R. H., & McArdle, R. (2007). Intra- and inter-session test, retest reliability of the Words-in-Noise (WIN) test. *Journal of the American Academy of Audiology, 18*(10), 813-825.
