# Supplementary material for: Reproducibility in Cognitive Hearing Research: Theoretical Considerations and Their Practical Application in Multi-Lab Studies
Source: Front Psychol. 2020 Jul 16;11:1590. doi: 10.3389/fpsyg.2020.01590 (PMC7378399; doi:10.3389/fpsyg.2020.01590)
Supplement: Supplementary file 2 [file Data_Sheet_2.pdf]

Supplementary Material

Supplementary Table 2

Supplementary Table 2. Examples of studies providing retest reliability coefficients for the same cognitive tests. HL = hearing loss; MMHL = mild-to-moderate hearing loss; HA = hearing aid users; NH = normal hearing; ICC = Intraclass Correlation Coefficient, PPMC = Pearson's Product-Moment Correlation; ? = information not provided

Test	Mode of administra-tion	Sample Size (N)	Age range	Group tested	Time between administrations (mean/median)	Type of test-retest coefficient	Value	Systematic difference between sessions (e.g., Practice / memory effects)	Reference
Digit Span (forward & backward)	Face-to-face	21	50 – 74<br>65.0 (mean)	Mild HL	4 weeks	ICC	0.88	No	Ferguson and Henshaw (2015)<br>Ferguson, Henshaw, Clark, and Moore (2014) for information on demography, design, practice effects
Digit Span forward	Face-to-face	24	50.1 (mean)	Hypertensives	7-10 days	PPMC	0.78	No	McCaffrey, Ortega, Orsillo, Nelles, and Haase (1992)
Digit Span backward		24					0.85	Yes	
Digit Span	Face-to-face	122	57 – 85<br>70.4 (mean)	Healthy	3 administrations, 1 year apart	?	0.64 (Y1-Y2)<br>0.64 (Y2-Y3)	?	Mitrushina and Satz (1991)
Letter Number Sequencing	Face-to-face	30	50 - 74<br>67.4 (mean)	MMHL, HA users	1 week	ICC	0.83	?	Ferguson and Henshaw (2015)<br>Henshaw and Ferguson (2014) for information on demography and design

Test	Mode of administra-tion	Sample Size (N)	Age range	Group tested	Time between administrations (mean/median)	Type of test-retest coefficient	Value	Systematic difference between sessions<br><br>(e.g., Practice / memory effects)	Reference
Letter Number Sequencing	Computeri-zed assessment	15	23.9 (mean)	Healthy	10-12 days	ICC	0.65	No	Vora, Varghese, Weisenbach, and Bhatt (2016)
Phonological fluency (letter S)	Face-to-face, 1min	99	39 - 75	Healthy	1 month	PPMC / Spearman	0.63	Yes	Bird, Papadopoulou, Ricciardelli, Rossor, and Cipolotti (2004)
Semantic fluency (animals)							0.56		
Phonological fluency 3 letters (letters F, A, S)	Face-to-face, 1min	90	Working age	Healthy	1 – 8 weeks	PPMC	0.82	?	Harrison, Buxton, Husain, and Wise (2000)
Phonological fluency letter B		90					0.73		
Semantic fluency (animal)		90					0.68		
Category naming (Semantic fluency)	Computeri-zed assessment, 1 min	15	23.9 (mean)	Healthy	10-12 days	ICC	0.78	No	Vora et al. (2016)
Trail Making A	Face-to-face	25	50. 1 (total group mean)	Hypertensives	7 – 10 days	PPMC	0.80	No	McCaffrey et al. (1992)
Trail Making B		24					0.92	No	
		32	59.1 (total group mean)	Chronic cigarette smokers			0.49	Yes	
Trail Making A	Face-to-face	122	57 – 85<br>70.4 (mean)	Healthy	3 administrations, 1 year apart	?	0.53 (Y1-Y2)<br>0.56 (Y2-Y3)	?	Mitrushina and Satz (1991)
Trail Making B							0.67 (Y1-Y2)<br>0.67 (Y2-Y3)		
Trail Making Test A	Computeri-zed assessment	16	18 – 34<br>24 (mean)	Healthy	3 administrations, 1 week apart	PPMC / Spearman	0.52 (W1-W2)<br>0.55 (W2-W3)	No	Palmer, Langbehn, Tabrizi, and Papoutsi (2018)
Trail Making Test B							0.76 (W1-W2)<br>0.66 (W2-W3)	No	

References

Bird, C. M., Papadopoulou, K., Ricciardelli, P., Rossor, M. N., & Cipolotti, L. (2004). Monitoring cognitive changes: Psychometric properties of six cognitive tests. *British Journal of Clinical Psychology, 43*, 197-210.

Ferguson, M. A., & Henshaw, H. (2015). How does auditory training work? Joined-p thinking and listening. *Seminars in Hearing, 36*(4), 237-249.

Ferguson, M. A., Henshaw, H., Clark, D. P., & Moore, D. R. (2014). Benefits of phoneme discrimination training in a randomized controlled trial of 50- to 74-year-olds with mild hearing loss. *Ear and Hearing, 35*(4), e110-121. doi:10.1097/AUD.0000000000000020

Harrison, J. E., Buxton, P., Husain, M., & Wise, R. (2000). Short test of semantic and phonological fluency: Normal performance, validity and test-retest reliability. *British Journal of Clinical Psychology, 39*, 181-191.

Henshaw, H., & Ferguson, M. A. (2014). *Assessing the benefits of auditory training to real-world listening: Identifying appropriate and sensitive outcomes.* Paper presented at the ISAAR 2013: Auditory Plasticity—Listening with the Brain. 4th Symposium on Auditory and Audiological Research, Nyborg, Denmark.

McCaffrey, R. J., Ortega, A., Orsillo, S. M., Nelles, W. B., & Haase, R. F. (1992). Practice effects in repeated neuropsychological assessments. *The Clinical Neuropsychologist, 6*(1), 32-42. doi:10.1080/13854049208404115

Mitrushina, M., & Satz, P. (1991). Effect of repeated administration of a neuropsychological battery in the elderly. *Journal of Clinical Psychology, 47*(6), 790–801.

Palmer, C. E., Langbehn, D., Tabrizi, S. J., & Papoutsi, M. (2018). Test-retest reliability of measures commonly used to measure striatal dysfunction across multiple testing sessions: A longitudinal study. *Frontiers in Psychology, 8*, 2363. doi:10.3389/fpsyg.2017.02363

Vora, J. P., Varghese, R., Weisenbach, S. L., & Bhatt, T. (2016). Test-retest reliability and validity of a custom-designed computerized neuropsychological cognitive test battery in young healthy adults. *Journal of Psychology and Cognition, 1*(1), 11-19.
